# Multi‐Plant Concentrated Powder Improved Skin Whitening: A Double‐Blinded, Randomized, and Placebo‐Controlled Clinical Study

**DOI:** 10.1111/jocd.70011

**Published:** 2025-02-10

**Authors:** Yuxiang He, Yongshi Bu, Chi‐Fu Chiang, Yung‐Hsiang Lin, Chia‐Hua Liang, Leong‐Perng Chan, Jiawen Sun

**Affiliations:** ^1^ Melaleuca (China) Wellness Products Co. Ltd Idaho Falls USA; ^2^ Research & Design Center, TCI Co. Ltd. Taipei Taiwan; ^3^ Department of Cosmetic Science and Institute of Cosmetic Science Chia Nan University of Pharmacy and Science Tainan Taiwan; ^4^ Department of Otorhinolaryngology‐Head and Neck Surgery, Faculty of Medicine, College of Medicine Kaohsiung Medical University Hospital, Kaohsiung Medical University Kaohsiung Taiwan; ^5^ Department of Otorhinolaryngology‐Head and Neck Surgery, Kaohsiung Municipal Ta‐Tung Hospital Kaohsiung Medical University Kaohsiung Taiwan

**Keywords:** antioxidants, plant extracts, skin aging, skin pigmentation

## Abstract

**Introduction:**

With the growing demand for skin‐enhancing products in the market, research into edible plants has expanded significantly. Numerous studies have shown that plant extracts rich in phytochemicals can effectively improve skin issues such as wrinkles, pigmentation, and dullness. However, clinical studies focusing on the effects of combined ingredients are still limited.

**Objective:**

This study combined three plant ingredients known for their skin‐beautifying effects and clinically tested their functional properties.

**Methods:**

Sixty healthy subjects were screened and randomly divided into two groups: a test group (TG) and a placebo group (PG). TG took two tablets of multi‐plant concentrated powder daily, while PG took placebo tablets without active ingredients. The study lasted 12 weeks. Plasma Trolox equivalent antioxidant capacity (TEAC), superoxide dismutase (SOD), and glutathione peroxidase (GPx) were measured at week 0 (W0), week 8 (W8), and week 12 (W12). Skin brightness (L* value), skin tone (Individual Typological Angle (ITA°)), erythema (a* value), skin spots, and wrinkles were assessed at week 0 (W0), week 4 (W4), week 8 (W8), and week 12 (W12).

**Results:**

The study showed significant improvements in all three antioxidant markers in the blood after consuming the compound ingredient tablets compared to the PG (*p* < 0.01). L* value and ITA° significantly increased from 8 weeks (*p* < 0.1). Skin spots significantly decreased at W8 and W12 (*p* < 0.1). While a* value and skin wrinkles showed noticeable reductions within the group in week 12 (*p* < 0.1), there were no significant differences compared to the PG.

**Conclusions:**

Consumption of multi‐plant concentrated powder improved skin whitening, brightened skin tone, reduced skin spots, and showed some improvement in wrinkles and erythema.

**Trial Registration:**

ClinicalTrials.gov identifier: NCT05988567

## 1 Introduction

The growing interest in skin brightening and anti‐pigmentation products has driven research into the mechanisms of melanin production in the skin. Human skin color is determined by the amount of melanin in the epidermis, produced by melanocytes [1]. Although the number of melanocytes is consistent across ethnicities, variations in skin color result from differences in melanin production and metabolism [2]. Melanin synthesis starts with tyrosine conversion to dopaquinone, catalyzed by tyrosinase, and continues through a series of reactions leading to melanin formation [3]. Ultraviolet radiation (UVR) is a key trigger for melanin production, inducing free radicals, oxidative stress, and increased melanin synthesis [4]. While melanin protects the skin, excessive production can cause pigmentation issues like freckles and hyperpigmentation [1]. Thus, identifying ingredients that inhibit tyrosinase activity or reduce UVR‐induced oxidative stress is essential for developing effective skin whitening solutions.

Melon rich in superoxide dismutase (SOD) plays a crucial role in neutralizing superoxide radicals generated by UV radiation [5]. SOD reduces oxidative stress, potentially preventing the activation of melanogenesis, the process of melanin production stimulated by oxidative damage [6]. Clinical studies have shown that melon concentrate enhances endogenous antioxidant enzyme activity, inhibits UV‐induced oxidative stress, increases the minimal erythema dose (MED), and provides photoprotective effects [7]. Olive fruit contains hydroxytyrosol, a powerful antioxidant that mitigates oxidative stress and lipid peroxidation [8]. By reducing oxidative stress, hydroxytyrosol may inhibit the pathways that upregulate tyrosinase, the enzyme responsible for melanin production, leading to decreased melanin synthesis and contributing to skin whitening [9]. Oral intake of olive fruit powder reduces lipid peroxidation and enhances skin's photoprotection [10]. Pomegranate rich in polyphenols and ellagitannins also exerts skin‐whitening effects by inhibiting tyrosinase activity and reducing melanin synthesis [11]. These compounds scavenge free radicals and lower oxidative stress, thereby preventing melanin overproduction [12]. Ellagitannins, in particular, combine antioxidant and anti‐tyrosinase activities, offering a dual approach to reducing pigmentation and achieving a more even skin tone [13].

Despite the individual benefits of these plant extracts, it remains uncertain whether a combined multi‐plant formulation (melon, olive fruit, pomegranate) provides superior skin benefits. This clinical study aims to explore and clarify the potential synergistic effects of this combination on skin health.

## 2 Materials and Methods

### 2.1 Test Formulations

The formulations for the test product and placebo are listed in Table 1. Aside from melon freeze‐dried concentrate powder, containing no less than 14 000 IU/g SOD, olive fruit powder with 10% hydroxytyrosol, and pomegranate concentrate powder with 30% Punicalagins (A + B) and Punicalins (A + B), other excipients serve as binders. The test product contains three compound ingredients which shows a certain color due to their properties. The PG was colored using pigments to make it look similar as TG after compression into tablets. Both formulations were coated with the same coating powder to ensure uniform appearance.

**TABLE 1:** Ingredients list of test formulation and placebo formulation.

Placebo formulation	Test formulation
Microcrystalline cellulose	Melon freeze‐dried concentrate powder
Magnesium stearate	Pomegranate concentrate powder
Pigment	Olive fruit powder
D‐Sorbitol	Microcrystalline cellulose
C‐Protective film coating	Magnesium stearate
/	C‐Protective film coating

### 2.2 Experimental Design and Ethics

This study utilized a randomized, placebo‐controlled, double‐blind design. All participants provided written informed consent prior to their involvement in the trial. The study protocol received approval from the Institutional Review Board of Taipei Antai Hospital (Protocol No. 23‐049‐B). Participants were recruited and underwent testing at the Department of Cosmetic Application and Management, Chia Nan University of Pharmacy and Science, Taiwan. The trial was conducted from April 6, 2023, to April 30, 2024.

### 2.3 Study Participants

Healthy volunteers aged 25–65 with ITA° values between 20° and 41° were eligible. Exclusion criteria included pregnancy or lactation, planning for pregnancy in the near future, history of psoriasis, eczema, atopic dermatitis, severe acne, or other chronic systemic diseases. Participants who had taken oral or topical corticosteroids or other anti‐inflammatory drugs within the past month, used products or medications affecting skin color such as glycolic acid, salicylic acid, or hydroquinone preparations within the past 2 months, used retinoid preparations or undergone chemical peels, laser treatments, or intense pulsed light therapy within the past 3 months, or had unavoidable prolonged sun exposure were also excluded. The trial recruited 60 participants because, under a significance level of *α* = 0.05, a statistical power of 80%, a difference of 0.7, and a SD of 0.95, the required sample size per group is approximately 30 participants.

### 2.4 Test Protocol

**FIGURE 1:**
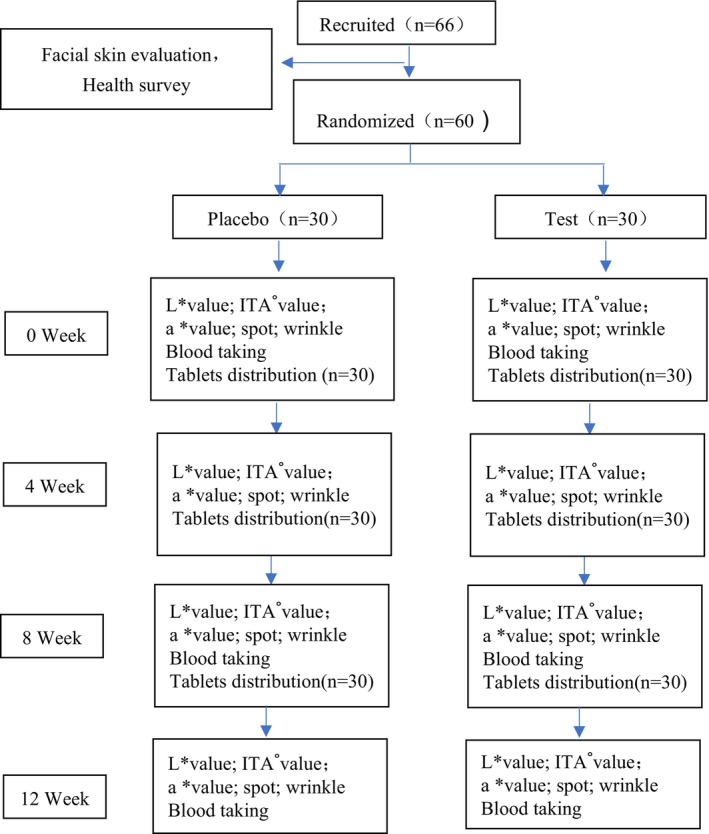
Study design and participant flow. The study enrolled 66 participants, of which 60 were randomized into two groups: The placebo group (*n* = 30) and the test group (*n* = 30). The study duration was 12 weeks, with assessments conducted at baseline (Week 0), Weeks 4, 8, and 12. Evaluations included skin parameters (L* value, ITA° value, a* value, skin spots, and wrinkles) as well as blood sampling for antioxidant markers.

During the initial screening, participants were selected based on skin type testing and health questionnaires. Before each assessment, participants cleansed their facial skin with water and sat quietly in a room at ambient temperature for 30 min. The testing facility was equipped with air conditioning set to 25°C and humidity maintained at 55% ± 5%. Test cheek for L* value, a* value, ITA°, and whole face for spots and wrinkles. Prior to starting the product regimen, baseline facial skin assessments (W0) and fasting blood collection after an 8‐h fast were conducted. After enrollment, participants were instructed to take 2 placebo tablets or test tablets daily after breakfast for a continuous 12 weeks. Follow‐up evaluations of facial skin were conducted at weeks 4, 8, and 12. Additionally, fasting blood samples were collected at week 8 and 12 (see Figure 1).

### 2.5 Test Items and Instruments

TEAC, SOD, and GPx levels were measured through blood tests. Skin fairness (L* value), ITA°, and erythema of the skin (a* value) were analyzed using a skin color difference analyzer (Chroma Meter MM500, Minolta, Japan). Skin pigmentation spots and wrinkles were assessed using the VISIA Complexion Analysis system (VISIA Complexion Analysis, U.S.A). The color of the skin is primarily determined by the amount of pigments such as melanin and erythema in the skin [7]. The ChromaMeter can measure L* value, a* value, and ITA°value. L* value represents brightness, with higher L* values indicating increased skin brightness. The a* value reflects the level of erythema, with lower a* values indicating reduced redness of the skin. ITA° is the most common indicator used to assess skin whitening effects. The definition of ITA° is as follows:

${\mathrm{ITA}}^{\circ }=\arctan\left(\frac{L^{*}-50}{b^{*}}\right)*\frac{180}{\pi }$

**FIGURE 2:**
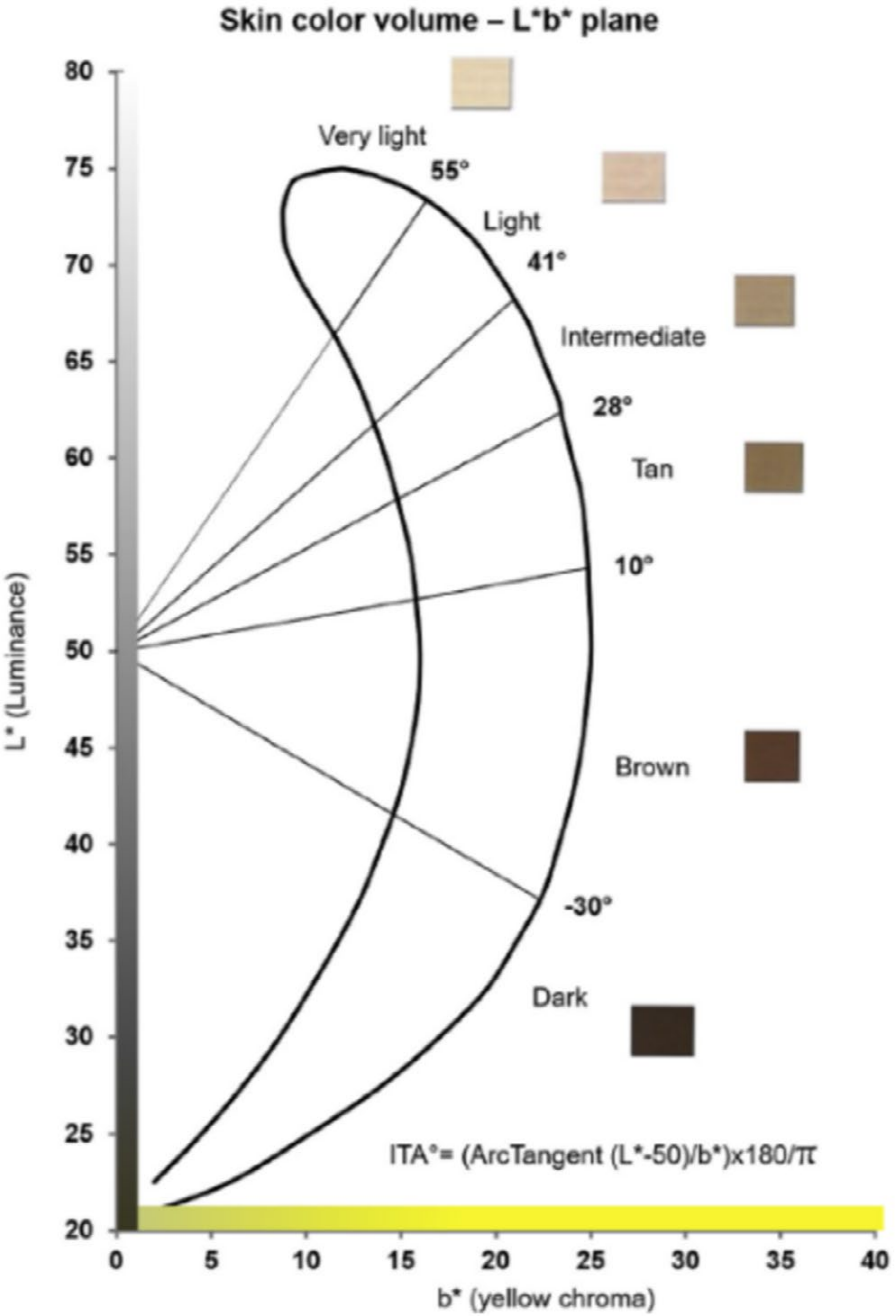
Skin color volume in the L*b*plane and individual typological angle (ITA°) classification. The graph illustrates the relationship between luminance (L*) and yellow chroma (b*) in skin color evaluation. The skin color categories are defined by ITA° values.

In this study, the skin tones of participants at baseline and the evaluation of whitening effects were assessed using the ITA° value. A higher ITA° value indicates lighter skin tone, while a lower ITA° value indicates darker skin tone. Refer to Figure 2 [8] for specific classifications.

### 2.6 Serum Biochemical Parameters

The fasting blood of each participant was collected at weeks 0, 8, and 12 for subsequent analysis of their physiological parameters. The blood samples were centrifuged at 2000 *r* for 15 min at 4°C. The clear serum samples were collected and stored at −80°C until tests were carried out. The values of the serum biochemical parameters including TEAC, SOD, and GPx were monitored. TEAC (TEAC Assay Kit; XAN‐5040), SOD (Superoxide Dismutase Assay Kit; BSKH62259), and GPx (GPx Assay Kit; Cayman BSKH61049) were analyzed according to the instructions of the manufacturers of the corresponding kits.

### 2.7 Statistical Analysis

The comparison of measurement results for the skin parameters among groups and between groups was analyzed by Student's *t*‐test and one‐way ANOVA, respectively, with R software. The data follow a normal distribution. Statistical significance is indicated as *, *p* < 0.05; **, *p* < 0.01; ***, *p* < 0.001 versus week 0 for the group, and ^#^, *p* < 0.05; ^##^, *p* < 0.01; ^###^, *p* < 0.001 versus placebo at week 0.

## 3 Results

### 3.1 Basic Information of the Participants

The treatment group (TG) comprised 30 participants, including 26 females and 4 males, with a mean age of 47.5 ± 10.1 years. The placebo group (PG) also consisted of 30 participants, including 25 females and 5 males, with a mean age of 48.5 ± 11.6 years. Throughout the study, no participants had withdrawn, and no product‐related adverse effects had been reported. Table 2 showed that baseline characteristics between the two groups had shown no significant differences.

**TABLE 2:** Baseline data between PG and TG.

Parameter	PG (0 week)	TG (0 week)	*p*
TEAC	227.8 ± 31.4	223.4 ± 41.4	0.143
SOD	6.4 ± 2.5	6.2 ± 2.5	0.667
GPx	504.1 ± 361.1	668.6 ± 555	0.180
L* value	57.2 ± 3.5	57.9 ± 1.8	0.280
ITA°	25.2 ± 12.6	28.0 ± 7.3	0.298
Spots	169.7 ± 46.5	153.5 ± 40.1	0.153
a* value	9.0 ± 2.0	8.7 ± 1.2	0.564
Wrinkles	55.7 ± 34.8	55.5 ± 38.2	0.986

Abbreviations: a* value, Skin Redness (Erythema Index); GPx, Glutathione Peroxidase; ITA°, Individual Typological Angle; SOD, Superoxide Dismutase; L* value, Skin Lightness (Luminance); TEAC, Trolox Equivalent Antioxidant Capacity.

### 3.2 Multi‐Plant Concentrated Powder Has Antioxidant Properties

**FIGURE 3:**
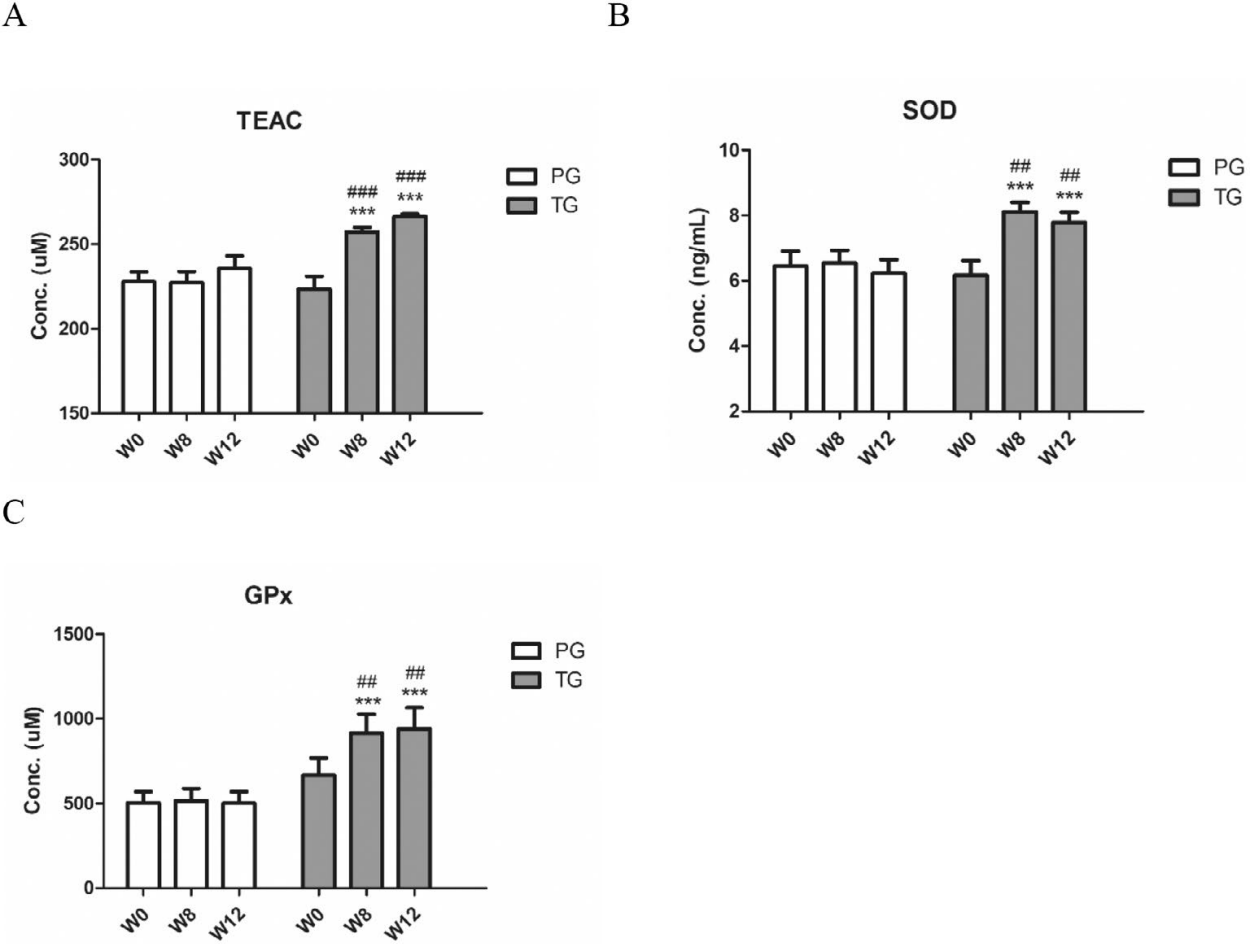
Multi‐plant concentrated powder can increase antioxidant capacity markers. (A) Trolox Equivalent antioxidant capacity (TEAC), (B) superoxide dismutase (SOD), and (C) glutathione peroxidase (GPx) levels were measured in the blood of participants at baseline (W0), week 8 (W8), and week 12 (W12). Compared to week 0: *, *p* < 0.05; **, *p* < 0.01; ***, *p* < 0.001; Compared to placebo: #, *p* < 0.05; ##, *p* < 0.01; ###, *p* < 0.001.

Figure 3 illustrated the changes in antioxidant capacity, measured by TEAC, in participants' blood after 12 weeks of supplementation with either compound ingredient tablets or placebo tablets. The results demonstrated that participants in the treatment group (TG) experienced a significant increase in TEAC levels at week 8 and 12, showing increases of 15.0% and 19.2%, respectively, compared to baseline (week 0). In contrast, the placebo group (PG) exhibited a decrease of 0.2% at week 8 and an increase of 3.5% at week 12, with no significant changes from baseline. Similar trends were observed for SOD and GPx levels, with significant increases in the TG at weeks 8 and 12, whereas the PG showed no significant changes in these antioxidant markers over the same period.

### 3.3 Multi‐Plant Concentrated Powder Can Improve Skin Condition

**FIGURE 4:**
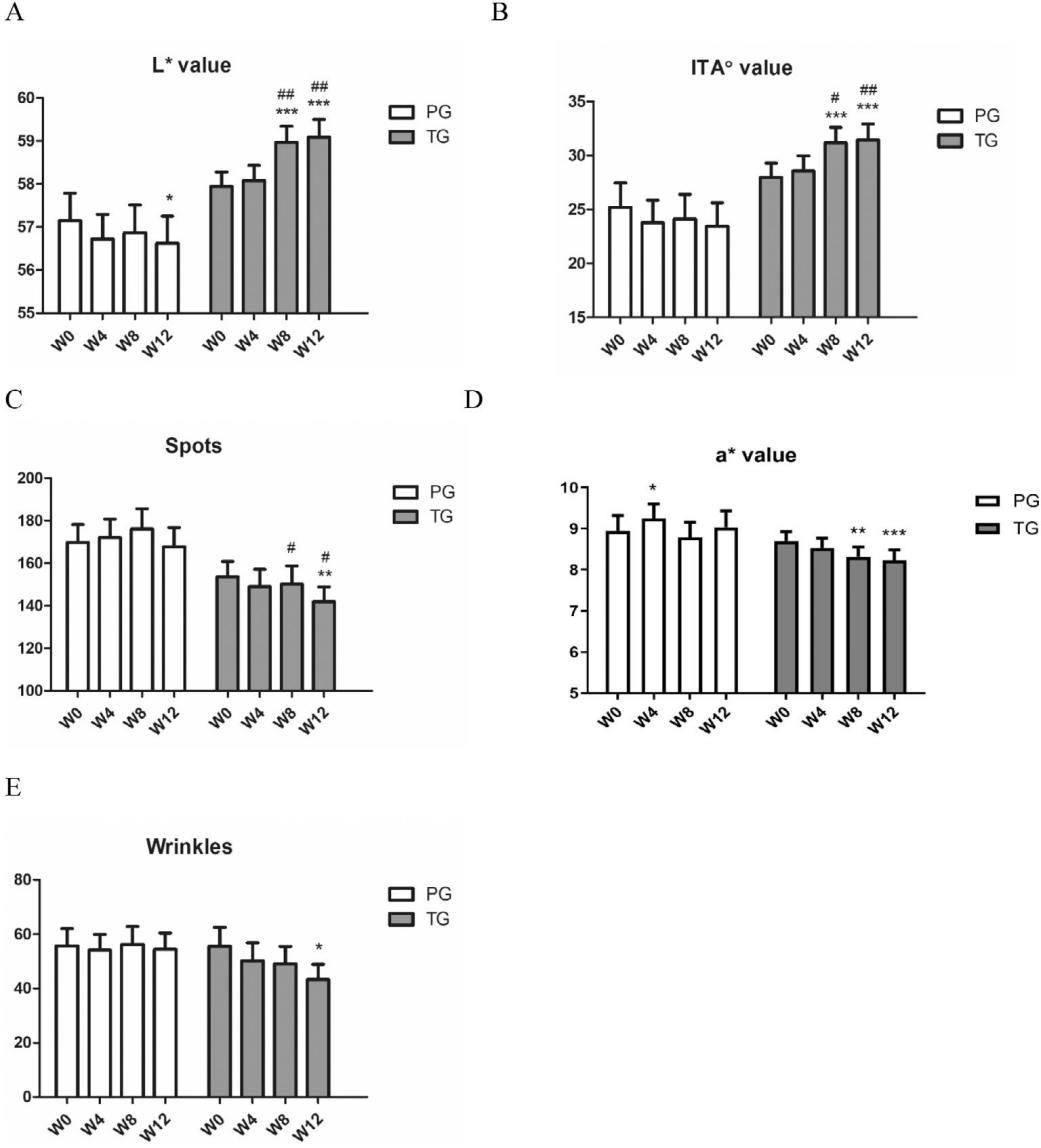
Multi‐plant concentrated powder can improve skin parameters. (A) L* value: Skin brightness measurements at weeks 0, 4, 8, and 12. (B) ITA° value: Skin brightness (ITA°) measurements at weeks 0, 4, 8, and 12. (C) Spots: Skin spot counts at weeks 0, 4, 8, and 12. (D) a* value: Skin redness measurements at weeks 0, 4, 8, and 12. (E) Wrinkles: Skin wrinkle measurements at weeks 0, 4, 8, and 12. Compared to week 0: *, *p* < 0.05; **, *p* < 0.01; ***, *p* < 0.001; Compared to placebo: #, *p* < 0.05; ##, *p* < 0.01; ###, *p* < 0.001.

**FIGURE 5:**
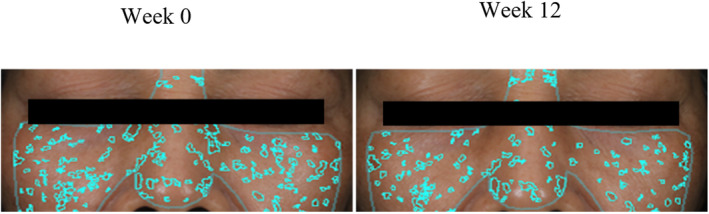
Multi‐plant concentrated powder can improve skin spot. VISIA images of skin spots at Week 0 and Week 12 in a subject from the treatment group (TG). Images show skin spot distribution before (Week 0) and after 12 weeks (Week 12) of supplementation with compound ingredient tablets. Spots are highlighted in blue for visualization.

**FIGURE 6:**
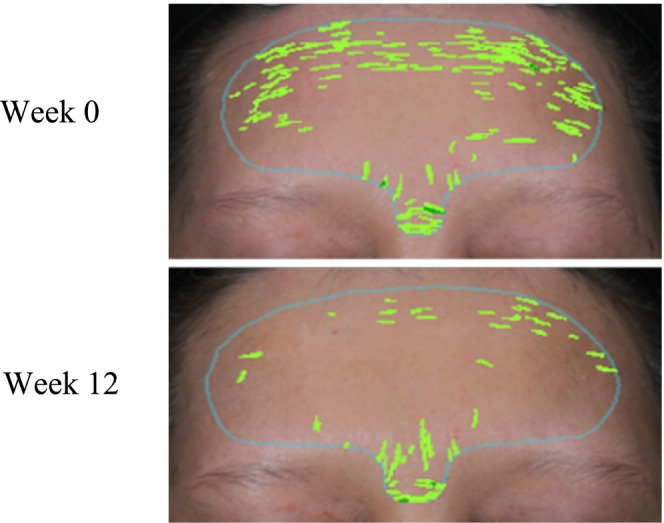
Multi‐plant concentrated powder can improve skin wrinkles. VISIA images of wrinkles at Week 0 and Week 12 in a subject from the treatment group (TG). Images show wrinkle distribution on the forehead before (Week 0) and after 12 weeks (Week 12) of supplementation with compound ingredient tablets. Wrinkles are highlighted in green for visualization.

In the TG, the skin brightness L* value significantly increased at week 8 and 12 (*p* < 0.001), with increases of 1.9% and 2.1% compared to week 0, respectively. Additionally, there was a significant difference compared to the PG (*p* < 0.01). The L* value in the PG significantly decreased by week 12, indicating that taking the compound ingredient tablets can help improve skin brightness (Figure 4A). After taking the compound ingredient tablets, the ITA° value, representing skin brightness, also significantly increased by 11.4% and 12.1% at week 8 and 12, respectively, compared to the PG, with significant differences observed. The PG experienced a slight decrease in skin brightness by week 12 (Figure 4B). After 12 weeks of taking the compound ingredient tablets, the subjects' skin spots significantly reduced by 7.6%, with a significant difference compared to week 0 (*p* < 0.01). The PG showed no significant differences throughout the test period. The TG had significantly fewer spots at week 8 and 12 compared to the PG (*p* < 0.05) (Figure 4C). The a* value, indicating skin redness, decreased by 4.6% and 5.7% at week 8 and 12, respectively, in the TG, with significant differences compared to week 0. The PG showed no significant changes (Figure 4D). In the TG, skin wrinkles decreased by 22.0% at week 12, with a significant difference compared to week 0, although there was no significant difference compared to the PG. Figure 5 shows VISIA images of a subject from the TG. At week 12, compared to week 0, there is a noticeable reduction in the number of skin pigmentation spots. Figure 6 depicts VISIA imaging of a subject from the TG. At week 12 compared to week 0, there is a notable reduction in the number of wrinkles.

## 4 Discussion

The clinical study found that taking the multi‐plant concentrated powder can improve skin whitening, brighten skin tone, reduce skin spots, and show some improvement in wrinkles and erythema. Additionally, the multi‐plant concentrated powder also possesses antioxidant properties, which help to protect the skin from oxidative stress and contribute to its overall anti‐aging effects.

Melon extract, particularly rich in the antioxidant enzyme superoxide dismutase (SOD), plays a crucial role in combating oxidative stress, a major contributor to skin aging and hyperpigmentation [14]. SOD helps reduce the production of reactive oxygen species (ROS), which can damage cellular structures such as DNA, lipids, and proteins, leading to skin dullness and dark spots [15]. Additionally, melon extract has been shown to inhibit tyrosinase, the enzyme responsible for melanin synthesis, thereby reducing melanin production and promoting a lighter skin tone with fewer dark spots [16]. Clinical studies have demonstrated that the application of melon extract improves skin brightness and tone by enhancing the skin's natural renewal process [17]. This promotes a more even distribution of melanin, resulting in a brighter complexion. The extract's anti‐inflammatory properties, likely mediated through the inhibition of pro‐inflammatory cytokines, contribute to reducing erythema and improving skin smoothness [18]. Moreover, melon extract supports collagen synthesis and protects against collagen degradation, aiding in wrinkle reduction and skin elasticity enhancement [19]. The SOD‐rich freeze‐dried melon concentrate powder, used in some studies, retains its freshness longer due to the freeze‐drying process, which concentrates SOD levels. Encapsulation of this powder ensures that active SOD reaches the intestines, overcoming the inactivation caused by stomach acid [20]. In animal models, encapsulated melon powder significantly increased plasma SOD levels and other antioxidant markers such as GPx and catalase [21]. In addition to SOD, the melon concentrate is rich in vitamins A and C, which further enhances its antioxidant properties, reducing free radical damage and minimizing pigmentation and spots [22], consistent with our results, that is, increase in antioxidant capacity and overall skin condition improvement.

Olive fruit is a rich source of polyphenols, particularly oleuropein, which transforms into hydroxytyrosol as the fruit matures [23]. Hydroxytyrosol, a powerful antioxidant, surpasses oleuropein in its ability to neutralize reactive oxygen species (ROS), which are major contributors to oxidative stress and skin aging [24]. Hydroxytyrosol inhibits tyrosinase, the enzyme responsible for melanin production, leading to reduced melanin synthesis [25]. Research indicates that oral intake of olive fruit extract significantly increases the minimal erythema dose (MED), extending the skin's tolerance to UV exposure and providing photoprotective effects [26]. This photoprotection is crucial in preventing UV‐induced skin damage and maintaining a healthy, youthful complexion. Daily consumption of hydroxytyrosol has been shown to elevate oxidative stress biomarkers like thiols and total antioxidant status (TAS) while reducing harmful compounds such as nitrates, nitrites, and malondialdehyde (MDA) [27]. It also inhibits elastase and collagenase activity, enzymes responsible for the breakdown of skin structure, thus preserving collagen and maintaining skin elasticity [28]. Hydroxytyrosol's ability to modulate the NF‐κB pathway reduces the production of pro‐inflammatory cytokines, minimizing skin redness (erythema) and promoting a smoother complexion [29]. Additionally, in skin fibroblasts exposed to UVA radiation, hydroxytyrosol reduces aging markers like β‐galactosidase activity and decreases the expression of matrix metalloproteinases (MMP‐1 and MMP‐3) [30], further highlighting its anti‐aging effects. Consistent with our results, the olive fruit in the formula increased antioxidant capacity and improved skin condition.

Pomegranates are rich in tannin polyphenols, including ellagic acid (EA), punicalagin (PC), and other pomegranate polyphenols (PG), which have demonstrated significant skin benefits [31]. Research indicates that these tannins, particularly punicalagin, ellagic acid, and gallic acid, can inhibit the formation of advanced glycation end‐products (AGEs), which are key contributors to skin aging [32]. These compounds exhibit anti‐glycation activity by scavenging carbonyl radicals, thus protecting the skin from damage associated with glycation. Pomegranate concentrate powder has also been shown to inhibit tyrosinase activity, thereby reducing melanin production [11]. This effect is achieved by suppressing the p38 and PKA signaling pathways, which are crucial in melanin synthesis [33]. Additionally, studies on pomegranate peel extract have demonstrated its ability to inhibit UV‐induced pigmentation in guinea pig skin by reducing the number of dopa‐positive melanocytes [11]. This suggests that pomegranate extract may achieve skin whitening by inhibiting both melanocyte proliferation and tyrosinase synthesis within melanocytes. Moreover, studies involving a combination of plant extracts from pomegranate (punicalagin), olive (hydroxytyrosol), and cistus (myricetin) have highlighted their collective skin‐whitening and anti‐UV effects [34]. These extracts not only reduce tyrosinase activity and melanin production but also repair UV‐induced damage to type I collagen and elastin, significantly reducing lipid peroxidation and protein glycation [28]. These findings support the whitening, antioxidant, and anti‐aging properties of pomegranate extract. Consistent with our results, the pomegranate extract in the formula increased antioxidant capacity and improved skin condition.

The limitation of this study was that, due to the use of a compound formulation, it was unclear whether the observed effects were attributable to a single active ingredient or the synergistic action of multiple ingredients. Further research was needed to confirm these mechanisms, and additional study groups were required for more comprehensive comparisons.

## 5 Conclusions

The clinical study confirmed that the multi‐plant concentrated powder enhances skin whitening, brightens skin tone, reduces spots, and improves wrinkles and erythema, largely due to its antioxidant properties. Future research should focus on clarifying the mechanisms behind these effects and determining whether they stem from individual ingredients or their synergistic interactions. Expanding clinical trials and comparisons with other treatments will further validate its potential for skincare applications.

## Author Contributions

Y.H., Y.B., J.S., and C.‐F.C. designed the research study. Y.‐H.L. provided resources, Y.H. and C.‐F.C. drafted the original paper, and L.‐P.C. and J.S. reviewed and edited the paper. C.‐H.L. and L.‐P.C. performed the research.

## Ethics Statement

The study protocol received approval from the Institutional Review Board of Taipei Antai Hospital (Protocol No. 23‐049‐B).

## Conflicts of Interest

The authors declare no conflicts of interest.

## Data Availability

Data sharing not applicable to this article as no datasets were generated or analysed during the current study.
